# Therapeutic Down-Modulators of Staphylococcal Superantigen-Induced Inflammation and Toxic Shock

**DOI:** 10.3390/toxins2081963

**Published:** 2010-07-29

**Authors:** Teresa Krakauer

**Affiliations:** Department of Immunology, Integrated Toxicology Division, United States Army Medical Research Institute of Infectious Diseases, 1425 Porter Street, Fort Detrick, Frederick, MD 21702-5011, USA; Email: Teresa.krakauer@amedd.army.mil; Tel.: +1-301-619-4733; Fax: +1-301-619-2348

**Keywords:** staphylococcal superantigens, therapeutics, inflammatory mediators, toxic shock, murine models

## Abstract

Staphylococcal enterotoxin B (SEB) and related superantigenic toxins are potent stimulators of the immune system and cause a variety of diseases in humans, ranging from food poisoning to toxic shock. These toxins bind directly to major histocompatibility complex (MHC) class II molecules on antigen-presenting cells and specific Vβ regions of T-cell receptors (TCR), resulting in hyperactivation of both monocytes/macrophages and T lymphocytes. Activated host cells produce massive amounts of proinflammatory cytokines and chemokines, activating inflammation and coagulation, causing clinical symptoms that include fever, hypotension, and shock. This review summarizes the *in vitro* and *in vivo* effects of staphylococcal superantigens, the role of pivotal mediators induced by these toxins in the pathogenic mechanisms of tissue injury, and the therapeutic agents to mitigate the toxic effects of superantigens.

## 1. Staphylococcal Exotoxins as Superantigens

### 1.1. Overview

*Staphylococcus aureus*, a ubiquitous gram-positive coccus, produces several exotoxins: staphylococcal enterotoxins A through R (SEA-SER), and toxic shock syndrome toxin 1 (TSST-1), which contribute to its ability to cause disease in humans and laboratory animals [1,2,3,4,5,6,7,8]. These exotoxins bypass the normal mechanism of conventional antigen processing, bind outside the peptide-binding groove of major histocompatibility complex (MHC) class II molecules on antigen-presenting cells (APC) and specific Vβ regions of T-cell receptors (TCR), to polyclonally activate T cells [2,9]. Originally known for their pyrogenicity, these bacterial toxins are called “superantigens” because of their potency in stimulating T cells at picomolar concentrations [2,9]. The dual affinity of staphylococcal superantigens for MHC class II molecules and specific TCR Vβ chains enables these microbial toxins to perturb the immune system and induce high levels of proinflammatory cytokines, chemokines, tissue factor, lytic enzymes, and reactive oxygen species, activating both inflammatory and coagulation pathways [10,11,12,13,14,15,16,17,18,19]. Important inflammatory cytokines, tumor necrosis factor α (TNFα) and interleukin 1 (IL-1) appear early, and in conjunction with other cytokines and chemokines they elicit fever, hypotension, and shock [20]. Interferon (IFN)γ, a prominant T-helper type 1 (Th1) cytokine, acts synergistically with TNFα and IL-1 to promote immune reaction and tissue injury. IL-2, from superantigen-activated T cells, causes vasodilation and contributes to vascular leak and edema [21]. The chemokines, IL-8, monocyte chemoattractant protein-1 (MCP-1), macrophage inflammatory protein 1α (MIP-1α), and MIP-1β, are induced directly by SEB or TSST-1 and selectively activate and direct migration of leukocytes, neutrophils and dendritic cells to sites of tissue injury [16,17]. These mediators are pathogenic at high concentrations *in vivo* and induce fever, organ dysfunction, and death.

### 1.2. Physical properties of staphylococcal superantigens

Staphylococcal enterotoxins (SEs) and TSST-1 are 22 to 30 kD single-chain globular proteins with well-conserved tertiary structures [22]. Based on amino acid sequence alignment, staphylococcal superantigens can be grouped into three subfamilies [6,7,8,23]. SEA, SED, SEE, SHE, and SEI share the highest sequence homology, between 53% and 81%. The second group is comprised of SEB, the SECs, and SEG, which are 50% to 66% homologous. Finally, TSST-1 has only 28% identity with the rest of the SEs as it has a distinct, shorter primary sequence of 194 amino acids with no cysteines and a missing "disulfide loop" commonly found in SEs. This disulfide loop has been proposed to be associated with the emetic properties of SEs, as mutation of residues in this loop eliminated the emetic effects of SEC1. Crystallographic studies of staphylococcal superantigens reveal similarities in the secondary-tertiary structure with two conserved, tightly packed domains. The *N*-terminal β-barrel domain resembles the oligosaccharide/oligonucleotide-binding (OB) fold found in other unrelated proteins, while the *C*-terminal domain has a β-grasp motif [22]. The relatively conserved TCR-binding site is located in the shallow groove between these two domains [22,24].

### 1.3. Human diseases caused by staphylococcal superantigens

SEB is the prototypic and most widely studied superantigen and is listed by the Centers for Disease Control and Prevention (CDC) as a category B priority agent because it can be used as an air-borne, food-borne, and water-borne toxic agent. These bacterial toxins have profound effects on the immune system through the action of proinflammatory cytokines which affect local and distant sites of infection. Depending on the dose and route of exposure, SEB and other SEs cause food poisoning, acute and fatal respiratory distress, autoimmune diseases, and toxic shock [3,7,11,25].

Staphylococcal toxic shock syndrome is characterized by fever, hypotension, desquamation of skin, fever, and dysfunction of three or more organ systems [1]. Microbial superantigens are also causative agents of autoimmune diseases, as shown in several animal models by their ability to activate APC and normally quiescent, autoreactive T- and B- cells [26,27,28,29]. In humans, there is a good correlation between the presence of SEB-specific IgM and arthritis, suggesting a role for this toxin in disease [30]. TSST-1 may also be associated with Kawasaki syndrome, a disease with immunoregulatory abnormalities [31,32]. Psoriasis and atopic dermatitis are autoimmune diseases also linked to staphylococcal and streptococcal colonization of skin and subsequent production of exotoxins like SEA, SEB, SEC, TSST-1, and streptococcal pyrogenic exotoxins [3,29]. 

## 2. Superantigen Binding to Host Cells

### 2.1. Binding to MHC class II

Superantigens bind to common, conserved elements of MHC class II molecules with relatively high affinity (K_d_ = 10^−8^–10^−7^ M) [33]. However, each individual toxin exhibits preferential binding to certain MHC isotypes, allowing for different modes of contact for superantigen with MHC class II [33,34,35,36]. HLA-DR binds SE and TSST-1 better than HLA-DP or -DQ, and murine IE molecules bind better than IA [37,38]. Crystallographic analysis indicates two distinct sites on MHC class II molecules for superantigen binding. A common, low-affinity binding site is found on the invariant α-chain of MHC class II and a high-affinity, zinc-dependent binding site is located on the polymorphic β-chain [8,22,39,40,41]. Superantigens in the SEA subfamily can bind to both α and β chains of MHC class II, interacting with the OB fold and β-grasp domain, respectively. This mode of superantigen and MHC class II interaction enables the toxin to bind to both sides of the molecule and cross-link MHC class II on antigen-presenting cells (APC) [40]. Cross-linking allowed SEA to persist on the surface of APC and prolonged their exposure and effects on T cells [42]. SEB and TSST-1 bind only to the generic low-affinity site using the solvent-exposed, hydrophobic core at the *N*-terminal of the superantigen [38,39].

### 2.2. Binding to TCR

The interaction of each toxin with the TCR Vβ chain is unique, as shown by the different Vβ specificities of each superantigen [2,3,9]. Members of the same subgroup interact and stimulate a different but overlapping TCR Vβ. For example, SEB stimulates human T cells bearing Vβ 1.1, 3.2, 6.4, 15.1, while another member of this group, SEC1, binds T cells with Vβ 3.2, 6.4, 6.9, 12, 15.1 [8]. The binding contacts are mostly between the side-chain atoms of the superantigen and the complementarity-determining regions 1 and 2 and the hypervariable region 4 within the Vβ chain. Superantigens bind TCR Vβ with low affinity (K_d_ = 10^−4^–10^−6^ M), similar to those of conventional MHC/peptide/TCR Vβ interactions. The mitogenic potency of these toxins results from a cooperative interaction such that the superantigen/MHC complex binds the TCR with a higher affinity than toxin alone.

### 2.3. Co-stimulatory molecules on host cells

As with conventional antigens, optimal cell activation requires the expression of co-stimulatory molecules on APC and T cells. These co-stimulatory molecules provide potent signals in addition to TCR engagement and influence T cell differentiation into major subsets of T cells, Th1, Th2, and Th17 cells. Intercellular adhesion molecule (ICAM) on an APC promotes stable cell conjugate formation and is a key co-stimulatory signal and adhesion molecule for many cell types [43]. A requirement for IL-2 induction from superantigen-stimulated T cells is the activation of the CD28-regulated signal transduction pathway [44]. Co-stimulatory pairs of LFA-1/ICAM-1, CD80/CD28 and CD40/CD154 on APC and T cells contribute to modulating immune responses to both conventional antigens and superantigens [43,44,45,46,47]. Other cell surface molecules besides CD11a/ICAM-1, such as CD2 and ELAM, are also required for optimal activation of endothelial cells and T cells by SEB [45].

## 3. Immune Activation

### 3.1. Signal transduction

**Figure 1:**
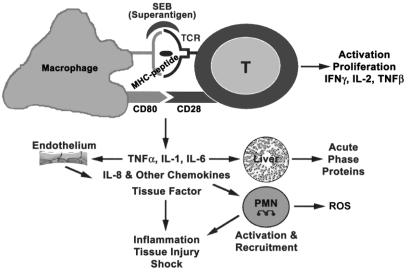
Cells and mediators participating in superantigen-induced toxic shock. Reproduced with permission from [54].

The main target cells of superantigens are the CD4^+^ T cells and mononuclear phagocytes bearing MHC class II molecules [2,48,49,50,51]. Engagement of superantigen with MHC class II and TCR on APC and T cells, respectively, activates intracellular signaling through receptor clustering. In T-cell clones, phosphatidyl inositol production and intracellular Ca^2+^ flux were early responses elicited by high concentrations of SEB [51]. Similar to mitogens and other cross-linking ligands of MHC class II, superantigens also activate protein kinase C (PKC) and protein tyrosine kinase (PTK) pathways [52,53]. Ultimately, activation of transcriptional factors NF-κB and AP-1 by superantigens results in the expression of proinflammatory cytokines, chemokines, and adhesion molecules on macrophages and T cells [51,53]. Additionally, the mediators produced by these superantigen-activated cells exert potent effects on the immune and cardiovascular system, resulting in multi-organ dysfunction and lethal shock (Figure 1) [54]. 

### 3.2. *In vitro* cellular response

Human peripheral blood mononuclear cells (PBMC) have been used extensively to study the cellular requirements for activation by staphylococcal superantigens, as these cells are sensitive to nanomolar concentrations of toxins. Superantigen-activated PBMC secrete the cytokines IL-1, IL-2, IL-6, IL-12, TNFα, TNFβ, IFNγ; the chemokines, IL-8, MCP-1, MIP-1α, MIP-1β [10,11,12,13,14,15,16,17,18]. Both monocytes and T cells are required for optimal induction of mediators as cognate interaction of superantigen bound on APC with T cells contributes to the production of these cytokines and chemokines [14,17,48,49]. Most of the mediators are induced as early as 5 h and are present as late as 72 h, whereas superantigen-induced T cell proliferation appears later, reaching maximum levels at 48 to 72 h. Direct superantigen presentation to T cells in the absence of MHC class II molecules can induce an anergic response [55]. 

Other cell types responding directly to staphylococcal superantigen include synovial fibroblasts, B cells, mast cells, intestinal myofibroblasts, intestinal and vaginal epithelial cells [56,57,58,59]. Superantigen-activated synovial fibroblasts triggered chemokine gene expression, raising the possibility that superantigens can be a causative agent for inflammatory arthritis [57]. Internalized SEB was found in lysosomal compartments of human B cells [42] whereas in an intestinal epithelial cell line, transcytosis of SEB across the cell was observed [58]. The interactions of most superantigens with epithelial and endothelial cells/cell lines are mostly indirect, via the release of IL-1, TNFα, and IFNγ from superantigen-activated APC and T cells [60,61]. *In vivo*, SEB was shown to penetrate the gut lining, evoking local and systemic immune response [62]. Vaginal epithelial cells were shown to bind a dodecapeptide of amino acids conserved in all superantigens [59]. In response to SEA but not SEB, human intestinal myofibroblasts elicited IL-6, IL-8, and MCP-1 [63]. 

### 3.3. Signaling and biological effects of proinflammatory mediators

TNFα and IL-1 have overlapping and similar biological effects. Each can induce the production of other cytokines, chemokines, and cell adhesion molecules with potent immunological and vascular effects [20]. These two proinflammatory cytokines each activate the transcription factor NF-κB independently in many cell types that include epithelial and endothelial cells and synergizes with IFNγ to enhance immunological reactions. The individual and synergistic action of these three cytokines increases the expression of MHC class II, adhesion molecules, and tissue factor on endothelial cells, resulting in disseminated intravascular coagulation. On epithelial cells, these cytokines decrease trans-epithelial resistance and increase ion fluxes and protein transport. The receptors, adaptors, and the signaling molecules used by these three cytokines are vastly different and represent three families of cytokine receptors. 

**Figure 2:**
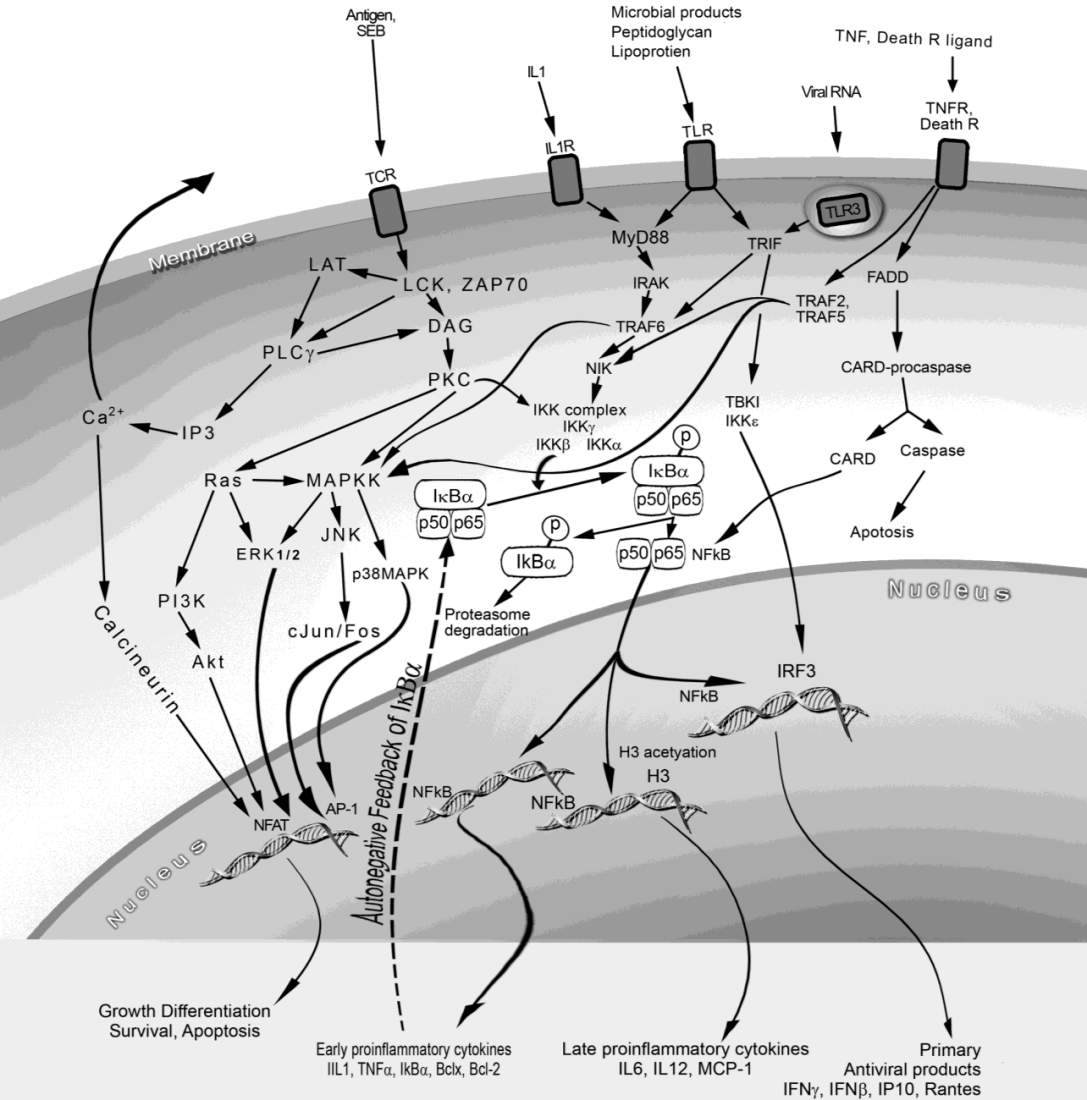
Cell receptors, intracellular pathways and signaling molecules used by superantigens and mediators induced by superantigens.

IL-1, an endogenous pyrogen, interacts with IL-1 receptor 1 (IL-1R1) subsequently activating downstream signaling molecules: the adaptor myeloid differentiation factor (MyD88), IL-1R-associated protein kinase (IRAK), and TNF receptor-associated factor 6 (TRAF-6) (Figure 2) [20]. Although toll-like receptors (TLRs) are not used for superantigen signaling, there are many similarities between the intracellular adaptors and signaling molecules used by the IL-1R and TLRs. The TLRs are conserved type 1 transmembrane receptors used by pathogen associated or released molecules to stimulate host innate immune responses [64]. Lipoproteins from gram-negative bacteria and lipopolysaccharide (LPS) from gram positive bacteria use TLR2 and TLR4, respectively, to activate NF-κB through the MyD88-dependent pathway, activating downstream IκB kinases (IKK). The phosphorylation of IκBα by IKK releases it from p50 and p65 of NF-κB, allowing for the translocation of NF-κB to the nucleus where it binds to promoter regions of many inflammatory genes [65,66]. Activation of NF-κB leads to induction of proinflammatory genes as well as antiapoptotic genes. 

TNFα and TNFβ, bind to TNF receptors (TNFR) and use intracellular TRAFs different from those used by IL-1R or TLR, but ultimately activating NF-κB, resulting in expression of cytokines and co-stimulatory molecules [20,67]. However, the TNFR possesses death domains, commonly present in receptors of the TNFR family, and binding of TNF to TNFR also triggers cell death through caspase activation. IFNγ binds to IFNγR, has many immunomodulatory properties different from IL-1 and TNF, but synergizes with these two cytokines in enhancing immune reactions [68]. IL-6 has some overlapping activities with IL-1 and TNFα [69]. Together and individually, these three cytokines act on the liver to release acute phase proteins, activate apoptopic pathways and decrease liver clearance function. 

Various chemokines from superantigen-activated cells attract neutrophils, T cells, dendritic cells, monocytes, and other cell types through seven-transmembrane G-protein-coupled receptors, induce early Ca^++^ flux, and activating phospholipase Cβ [20,70]. Cytokine- and chemokine-activated neutrophils, sequestered to sites of tissue injury and inflammation, produce reactive oxygen species (ROS) and proteases contributing to organ dysfunction. This is particularly important in the lung where systemic administration of SEB caused acute lung injury characterized by increased expression of adhesion molecules ICAM-1 and VCAM, increased neutrophils and mononuclear cells infiltrate, endothelial cell injury, and increased vascular permeability [71]. Intranasal delivery of SEB induced a prolong lung injury which was evident even after four days of steroid treatment [72]. Exudates from superantigen-injected air pouches were predominantly neutophils with some macrophages [16]. Endothelial cells surrounding air pouches expressed ICAM-1, TNFα, MIP-2 (an IL-8 related protein in mice), MIP-1α, and JE. Thus, systemic release of inflammatory mediators affects multiple organs and cell types resulting in decreased peripheral vascular resistance, multi-organ failure, hypotension, and shock. *In vivo*, SEB induced Vβ8-specific T cell proliferation but these cells were rapidly deleted in the periphery by Fas-FasL-dependent apoptosis [73]. 

## 4. Animal Models

### 4.1. Host response, administration route and dose effects

Microbial infections are complex diseases where invading pathogens must establish mechanisms to evade host detection and innate inflammatory responses for survival. Bacterial superantigens are among one group of virulence factors released by microbes and contribute to septic complications during infection [74,75]. Toxic shock syndrome represents a spectrum and progression of clinical features including multi-organ failure in humans exposed to bacterial toxins and/or concurrent bacterial infection, pathogenic features seemingly absent in most mouse models. In addition, humans are more sensitive to SEB intoxication and low doses are enough to cause lethal shock without the use of synergistic agents. In non-human primates, inhalation of lower, sublethal nanogram doses of aerosolized SEB caused severe incapacitation. TNFα was completely absent, although IL-2 and IL-6 were found in sera of this incapcatitation nonhuman primate model [76]. Microgram levels of aerosolized SEB in monkeys resulted in emesis and diarrhea that developed within 24 h of exposure, followed by the abrupt onset of lethargy, difficult breathing, hypotension, fever, and finally death from toxic shock [77]. Edema, leukocytic infiltration, and lesions were apparent in lung tissues in this model. The route of SE administration also influenced the pattern of cytokine expression. Intrathymic SEB administration induced a switch from Th1 to Th2 cytokine expression in the spleen [78]. Our own studies indicate that airway exposure was more potent in inducing lethality as lower intranasal (IN) doses can be used compared with just intraperiteonel (ip) injections [79]. 

### 4.2. Emetic response models

In humans and monkeys, SEs induced an emetic response and toxic shock at submicrogram concentrations [80]. Oral administration of SEB induces activation and expansion of murine Vβ8^+^ T-cells in Peyer’s patches, accompanied by increased IFNγ and IL-2 mRNA expression [81]. The enteric effects of SEs likely resulted from IL-2 release from activated T cells as IL-2 given to cancer patients produces side effects similar to staphylococcal food poisoning. The release of cysteinyl leukotrienes by mast cells and substance P from sensory neurons was responsible for emesis [56,82]. Recently, rapid release of serotonin and intestinal production of 5-hydroxytryptamine was seen in an emetic model using SEA [83]. Mice have no emetic response upon ingestion of high doses of SEs and are not suitable as emetic models.

### 4.3. Murine models of toxic shock using potentiating agents

Investigations of SEs in murine models of toxic shock have relied on potentiation agents to amplify the toxic effects of SEs as mice are poor responders to SEs due to lower affinity of these toxins to mouse MHC class II [84,85]. Sensitizing agents such as D-galactosamine (D-gal), actinomycin D, LPS, or viruses are used together with superantigens to induce toxic shock [84,85,86,87,88,89,90,91]. Depending on the injury model and adjunctive agents used, severity of disease may involve different organs and different pattern of mediators. Both D-gal and actinomycin D induced TNFα-dependent hepatotoxicity and SEB-induced shock models using these agents encountered much higher levels of TNFα not present when SEB was used alone and liver dysfunction was a prominent feature in these models. IL-2 deficient mice were resistant to SEB-induced shock in D-gal sensitized mice demonstrating the importance of IL-2 besides TNFα [92]. Antibodies to IFNγ inhibited SEB-induced weight loss and hypoglycemia but had no effect on mortality [93]. 

LPS, a cell wall component of gram negative bacteria, is the most frequently used potentiating agent in mouse models of SEB-induced shock. In these models, relatively high doses of SEB were administered via the aerosol, IN or i.p. route, followed by i.p. injections of LPS to induce shock [84,94,95,96,97]. LPS naturally synergizes with superantigens to induce the proinflammatory cytokine cascade and a correlation was found between increased serum levels of IL-1, IL-2, TNFα, and IFNγ with SEB-induced shock [84,94,95,96,97]. The shock syndrome induced by superantigens in these models results from the culmination of biological effects of much higher levels of IL-1, TNFα, and IFNγ, not seen when LPS was absent. It is difficult to determine if any one particular cytokine, and at what serum concentration, is required to cause SEB-induced toxic shock, as LPS induces the same proinflammatory cytokines and chemokines (TNFα, IL-1, IFNγ, IL-6, IL-8 and MCP-1) as superantigens. A recent study analyzed the interdependent effects of doses of SEB used alone and together with LPS in different combination of doses on serum levels of cytokine/chemokine in Balb/c mice, the most common mouse strain used in LPS potentiated SEB shock model [98]. *In vivo*, SEB alone induced only moderate levels of IL-2 and MCP-1 and all mice survived even with a high dose of SEB (100 µg/mouse). LPS (80 µg/mouse) alone caused 48% lethality and induced high levels of IL-6 and MCP-1. SEB induced low levels of TNFα, IL-1, IFNγ, MIP-2 and LPS synergized with SEB in the expression of these cytokines as well as those of IL-6 and MCP-1. Importantly, the synergistic action of SEB and LPS resulted in lethal shock and hypothermia not seen in SEB only or low doses of LPS. Cytokine levels by survival status in SEB plus LPS groups revealed significantly higher levels of TNFα, IL-6, MIP-2, and MCP-1 in non-survivors early after SEB administration. In addition to these cytokines and chemokines, significantly higher levels of IFNγ and IL-2 were observed at later times in non-survivors of toxic shock compared to those in survivors. In this LPS-potentiated SEB-induced shock model, the higher cytokine response, especially at the later time point, was influenced mostly by the LPS dose. Thus, the synergistic action of SEB and LPS promoted early TNFα release and prolonged the release of IL-6, IFNγ, IL-2, MIP-2, and MCP-1 in non-survivors. Overall, the higher as well as prolonged levels of these key cytokines led to acute mortality, with mice succumbing to toxic shock within 48 h when LPS was used together with SEB. Although there is a general agreement that Th1 cytokines, typified by IFNγ, are important in these potentiated models of SEB-induced shock, the role of Th2 cytokines cannot be overlooked. IL-10, a prototypic Th2 cytokine, was detected *in vivo* after repeated superantigen stimulation [99,100,101]. IL-10-deficient mice showed increase levels of IL-2, IFNγ, TNFα after SEB stimulation, and they were more susceptible to SEB-induced lethal shock [100]. Repeated superantigen exposure also generated immunosuppressive regulatory T cells with attendant IL-10 secretion and inhibited IL-2 production [102,103], accompanied by clonal deletion and apoptosis of some of these activated T cells [55,103]. 

### 4.4. Transgenic mouse models

The mechanism of SEB intoxication and therapeutic studies were also investigated using transgenic mice with human MHC class II [104,105,106,107]. Transgenics respond to much lower doses of toxins due to the higher affinity binding of SEs to human MHC class II molecules and high levels of serum IFNγ, IL-2, and IL-6 also correlated with mortality [106]. Although TNFα was present in lungs of HLA-DQ8 transgenics exposed to aerosolized SEB, serum TNFα was absent in this study [106]. Pathological lesions in lungs of transgenics, temperature fluctuations, lethality starting later at 96 h, were similar to those in nonhuman primates exposed to lethal doses of SEB. Other investigations [105] suggested that two doses of relatively high amounts of SEB (30 to 100 μg/mouse) were necessary to induce toxic shock in these transgenics, and the sensitizing agents D-gal was still required [107].

### 4.5. Murine models using only SEB

A high IN dose of SEB was reported to be lethal in C3H/HeJ, a TLR4-defective mouse strain, but the mechanism of intoxication was unclear [108]. A recent study revealed that this dose of SEB was ineffective in mediating SEB-induced shock, although two low doses of SEB, at least one dose must be delivered by IN, were lethal [79]. This two-hit model required two doses of SEB strategically given 2 h apart with the first SEB dose delivered by IN and the subsequent dose of SEB administered either IN or by i.p. Increased serum levels of IL-2, IL-6, and MCP-1 accompanied by an early, high concentration of lung MCP-1 was seen in this dual-dosing model [79]. MCP-1, a potent activator and chemotactic factor for T cells as well as monocytes probably contribute to early leukocyte recruitment into the lung in this IN SEB-induced shock model. The proinflammatory cytokines, IL-1, TNFα, and IFNγ were found in lungs but not in serum of SEB-exposed C3H/HeJ mice. Pathological lesions, temperature fluctuations, and time course of lethality also resembled those seen in transgenics, nonhuman primates, and humans [77,106]. 

## 5. Therapeutics for Superantigen-Induced Shock

### 5.1. Influence of animal models on efficacy of therapeutics

None of the existing mouse models completely reproduces all the complex events of human toxic shock syndrome as humans exposed to bacterial toxins often have concurrent bacterial infections. Each toxic shock model described in the previous section revealed the importance of different sets of cytokines and organ injury. In particular, the mouse models using potentiating agents such as actinomycin D, D-gal, or LPS created unrealistically higher levels of certain cytokines such as TNFα. As hepatic toxicity is TNFα-dependent, drugs designed to inhibit TNFα will have a higher therapeutic impact in models with TNFα-mediated toxicity. However, even nonhuman primate models for human disease in drug efficacy testing is no guarantee of reproducibility of therapeutic effectiveness as demonstrated by the cytokine storm elicited in phase 1 trial of the monoclonal antibody to CD28 [109]. 

Potential therapeutic targets to prevent the toxic effects of SEs include blocking the interaction of SEs with MHC or TCR, or other co-stimulatory molecules; inhibiting signal transduction pathways used by SEs; inhibiting cytokine and chemokine production; and blocking the downstream signaling pathways used by proinflammatory cytokines and chemokines.

### 5.2. Antibodies against superantigens

At present, intravenous immunoglobulins (IVIG) is the only therapeutic available for treating staphylococcal exotoxin-induced shock [110]. Antibody-based therapy targeting direct neutralization of SEB or other superantigens is most suitable at the early stages of exposure before cell activation and release of proinflammatory cytokines. Some of the neutralizing antibodies against one superantigen can cross-react and prevent the biological effects of a diifferent superantigen [111]. Vaccinations with recombinant mutants of SEB, which lack superantigenicity and had attenuated binding to MHC class II, were able to protect mice and monkeys against SEB-induced disease [112]. 

### 5.3. Inhibitors of cell receptor-toxin interaction

Since the binding regions of SEB to MHC class II and TCR are well-characterized, small overlapping peptides of SEB were used as antagonists to block the initial step of receptor-toxin interactions [113,114]. Conserved peptides corresponding to residues 150–161 of SEB prevented SEA-, SEB-, or TSST-1-induced lethal shock in mice when given intravenously 30 min after an i.p. toxin dose [113]. This segment of SEB is not associated with the classically defined MHC class II or TCR binding domains, but it likely blocks co-stimulatory signals necessary for T-cell activation. However, a subsequent study indicated that these peptides were ineffective inhibitors of SEB-induced effects both *in vitro* and *in vivo* [114]. Another recent study with yet a different peptide, dodecapeptide P72, did not bind MHC class II, but inhibited SEA, SEB, and SEC-mediated responses [115]. 

A different approach using a bi-specific chimeric inhibitor composed of the DRα1 domain of MHC class II and Vβ domain of the TCR specific for SEB connected by a flexible (GSTAPPA)_2_ linker bound SEB competitively and prevented its binding to MHC class II of APC and TCR on T cells [116]. These chimeric molecules blocked initial cellular activation and IL-2 release in SEB-stimulated PBMC. The drawback of this approach is that individual chimeras have to be constructed for each SE as TCR Vβdomains are different for each SE. Blockade of the CD28 co-stimulatory receptor by its synthetic ligand, CTLA4-Ig, prevented TSST-1-induced proliferation of T cells *in vitro* as well as lethal toxic shock *in vivo* [47]. Recently, polyphenols from apple juice were found to inhibit the biological activity of SEA *in vitro* and SEA was bound to constituents of apple juice [117].

### 5.4. Inhibitors of SEB signal transduction

Blockade of SEB signal transduction pathways represents a more amenable mode of intervention as these events are postexposure and will likely work for other SEs. NF-κB is an attractive therapeutic target as the activation of NF-κB leads to the inducible expression of many of the mediators involved in inflammatory diseases. *In vitro* and *in vivo* studies have shown that many of the genes (*i.e.*, cell adhesion molecules, co-stimulatory molecules, cytokines, chemokines, acute phase proteins, and inducible nitric oxide synthase) that are implicated in superantigen-induced lethal shock contain NF-κB binding sites in the promotor/enhancer region [65,66]. The nuclear import of NF-κB from cytoplasm upon phosphorylation and removal of IκBα allows transcriptional activation of over 100 genes that encode mediators of inflammatory responses and anti-apoptotic genes. Transient interruption of the NF-κB pathway may therefore be beneficial for superantigen-induced shock. A cell-penetrating cyclic peptide (cSN50) targeting NF-κB nuclear transport attenuated SEB-induced T cell responses and serum inflammatory cytokine [118]. Liver apoptosis and hemorrhagic necrosis and mortality were also reduced in mice given cSN50 before D-gal [119]. Administering cSN50 30 minutes prior to IN SEB in BALB/c mice reduced proinflammatory cytokines and chemokines in the bronchoalveolar space, attenuated neutrophil and monocytes infiltration to the lung and vascular injury [119]. Bortezomib, another NF-κB inhibitor, decreased SEB- induced serum cytokines and chemokines levels but had no effect on mortality and liver necrosis *in vivo* [120]. Another potent NF-κB inhibitor is dexamethasone, a well-known immunosuppressive drug used clinically to treat various inflammatory diseases. *In vitro*, dexamethasone potently inhibited staphylococcal exotoxin-induced T-cell proliferation, cytokine release, and activation markers in human PBMC [15,72,97,121]. *In vivo*, dexamethasone also significantly reduced serum levels of cytokines and protected mice from SEB-induced shock in the two-hit SEB-only model and the SEB plus LPS model [72,97]. Furthermore, dexamethasone attenuated the hypothermic response to SEB in both models of toxic shock and improved survival of mice by 100% even when administered after SEB. 

Other signal transduction inhibitors include those directed against protein kinase C (PKC) and protein tyrosine kinase. H7, a PKC inhibitor and genistein, a tyrosine kinase inhibitor each blocked TNFα but not IL-1 production from TSST-1-stimulated PBMC [122]. D609, an inhibitor of phospholipase C, which is an upstream activator of PKC, attenuated SE-induced effects both *in vitro* and *in vivo* [123,124]. Reduction in lethality was seen in mice treated with D609 and superantigen despite the unaltered high serum level of TNFα [124]. 

Recently, another FDA-approved immunosuppressive drug, rapamycin was shown to protect SEB-induced shock even when administered 24 h after SEB [125]. Rapamycin is used clinically to prevent graft rejection in renal transplantation, as it shows less nephrotoxicity than calcineurin inhibitors [126]. Rapamycin inhibited T cell cytokines and likely interfered with other T cell signaling pathway induced by SEB.

### 5.5. Inhibitors of cytokine induction

Most therapeutic testing in animal models of SEB-induced shock have targeted proinflammatory cytokines, as there is a strong correlation between toxicity and increased serum levels of these inflammatory mediators, particularly when SEB is used with potentiating agents [93,94,95,96,97]. Neutralizing antibodies against TNFα or soluble TNF receptor 1 prevented SEB-induced lethality, establishing the critical role of TNFα in SEB-induced shock [86,87]. The anti-inflammatory cytokine IL-10 blocked the production of IL-1, TNFα, and IFNγ, and reduced lethality to superantigen-induced toxic shock [101].

Another strategy is to attenuate IL-1 release from superantigen-activated cells is to target caspase 1, a proteolytic enzyme that cleaves pro-IL-1 into active IL-1 [20]. The caspase 1 specific inhibitor, Ac-YVAD-cmk, blocked IL-1 and MCP production in superantigen-stimulated PBMC cultures but had no effect on other cytokines or T-cell proliferation [127]. A pan-caspase inhibitor, Z-D-CH_2_-DCB, attenuated the production of IL-1β, TNFα, IL-6, IFNγ, MCP, MIP-1α, MIP-1β, and inhibited T-cell proliferation in SEB- and TSST-1-stimulated PBMC [127]. *In vivo*, pan-caspase inhibitor delayed the time of death in the SEB plus LPS model but was ineffective in preventing mortality (unpublished observations). Because IFNγ acts in synergy with IL-1 and TNFα, small amounts of IFNγ can profoundly affect the potentiation effects with IL-1 and TNFα [20]. *In vivo* neutralization of IFNγ either protects or harms the host, depending on the mouse model and dose of sensitizing D-gal used [93]. 

Other drugs tested to block cytokine release from superantigen-activated cells include doxycycline, an antibiotic, and pentoxyfylline, a methylxanthine derivative [95,128]. Doxycycline inhibited SEB-induced proinflammatory cytokines and chemokines and T-cell proliferation in human PBMC [128]. Pentoxyfylline, a phophodiesterase inhibitor, is used clinically to treat peripheral vascular disease. Its interference with intracellular pathways affects leukocyte adhesion and cytokine production. Pentoxyfylline inhibited SEB- or TSST-1-induced toxic shock, as well as cytokine and chemokine release [15,95]. Dexamethasone, pentoxyfylline, doxycycline, and rapamycin are FDA-approved drugs used for other indications, and have been in clinical use for many years with a proven safety record. A list of small molecular weight inhibitors effective in blocking SEB-induced shock is shown in Table 1.

**Table 1:** Small nonpeptide therapeutics for SEB-induced shock.

Pharmacologic agent	Target	Biological effects against SEB
Rapamycin FDA-approved for prevention of renal graft rejection	Immunophilin FK506BP12	Blocked SEB-induced MCP-1 and IL-6 *in vitro* and *in vivo* [125].
		Protected mice from lethality even when administered 24 h after SEB.
Dexamethasone FDA-approved for treating inflammatory diseases	NF-κB	Inhibited SEB-induced proinflammatory cytokines and chemokines in PBMC [72] and adhesion molecules (ICAM, ELAM, VCAM) on endothelial cells [121].
		Reduced serum levels of cytokines, attenuated hypothermia due to SEB, improved survival of mice [72,97].
Pentoxifylline FDA-approved for treating peripheral arterial disease	Phosphodiesterase	Attenuated SEB-induced proinflammatory cytokines and chemokines in PBMC [17,97].
		Blocked cytokine release *in vivo* and prevented SEB-induced lethal shock in SEB + LPS murine models [97].
Pirfenidone	Inhibition of TGFβ (exact mechanism unknown)	Inhibited SEB-stimulated cytokines *in vitro* and *in vivo* [96].
		Improved survival of mice [96].
Niacinamide	Nitric oxide synthase	Inhibited serum IL-2 and IFNγ [94].
		Prevented death of mice from SEB-mediated shock [94].
D609	Phospholipase C	Blocked SEB-stimulated cytokines, chemokines and proliferation in human PBMC [48].
		Improved survival of mice [103].

### 5.6. Inhibitors of cytokine signaling

Blocking the signal transduction pathways used by inflammatory cytokines represents yet another means of inhibiting cellular responses to superantigens. One target is the suppressor of cytokine signal 3 (SOCS3) which is an endogenous stop signal for IFNR-mediated responses and regulates the STAT family of proteins [68]. A cell-penetrating form of SOCS3 protected mice from the lethal effects of SEB and LPS by reducing production of inflammatory cytokines and attenuating liver apoptosis and hemorrhagic necrosis [129]. 

## 6. Summary

Proinflammatory cytokines act synergistically on multiple cells and organs resulting in cardiovascular derangement, multi-organ failure, and shock seen in SEB-induced diseases. The ability to stop the cytokine cascade and the inflammatory events initiated by cytokines early appears to be critical in preventing toxic shock. However, the tissue damage from cytokine storm lingers and resolution of inflammation, especially in the lungs, appears to be critical in preventing shock.

## Declaim

The views expressed in this publication are those of the author and do not reflect the official policy or position of the Department of the Army, the Department of Defense or the U.S. Government.
